# Simultaneous quantification of artesunate and mefloquine in fixed-dose combination tablets by multivariate calibration with middle infrared spectroscopy and partial least squares regression

**DOI:** 10.1186/s12936-016-1157-1

**Published:** 2016-02-24

**Authors:** Breno Maurício Marson, Raquel de Oliveira Vilhena, Camilla Regina de Souza Madeira, Flávia Lada Degaut Pontes, Mário Sérgio Piantavini, Roberto Pontarolo

**Affiliations:** Laboratório de Controle de Qualidade, Departamento de Farmácia, Universidade Federal do Paraná, 8021 Av Prefeito Lothário Meissner, 632, Curitiba, Paraná 80210-170 Brazil

**Keywords:** Artesunate, Mefloquine, DRIFTS, Multivariate analytical, PLS, Analytical validation, Quality control

## Abstract

**Background:**

Malaria is one of the most lethal and life-threatening infectious diseases in the world, causing more than half a million deaths annually. Treatment with mefloquine and artesunate is currently recommended by the World Health Organization, and was historically the first artemisinin-based combination therapy used clinically for treatment of *Plasmodium falciparum*. The problem of poor-quality medicines, such as counterfeit and sub-standard anti-malarials, is a worldwide issue; therefore, it is essential to develop rapid, low cost, solvent-free, and reliable methods for routine quality control for these drugs. The aim of this study was to develop and validate a novel multivariate method for direct simultaneous quantification of mefloquine and artesunate in tablets by diffuse reflectance, middle infrared spectroscopy and partial least squares regression (MIR-PLS).

**Methods:**

Diffuse reflectance infrared Fourier transform spectroscopy (DRIFTS) and partial least squares regression were applied for simultaneous quantification of artesunate and mefloquine in tablets provided by the Brazilian Government. The model was obtained with full spectra (4000–400 cm^−1^) preprocessed by first derivative and Savitzky-Golay smoothing followed by mean centring, and built with three latent variables. The method was validated according to Brazilian and international guidelines through the measuring of figures of merit, such as trueness, precision, linearity, analytical sensitivity, selectivity, bias, and residual prediction deviation. The results were compared to an HPLC–MS/MS method.

**Results:**

The MIR-PLS method provided root mean square errors of prediction lower than 2.0 mg per 100 mg of powder for the two analytes, and proved to be valid according to guidelines for analytical methods that use infrared (IR) spectroscopy with multivariate calibration. For the samples obtained from Brazilian healthcare units, the method provided results statistically similar to those obtained by HPLC–MS/MS.

**Conclusion:**

MIR-PLS was found to be suitable for the quality control of these drugs. It is fast, does not use solvents, and does not generate chemical waste. Furthermore, the proposed method may be transferred and developed for use in portable equipment, increasing the possibilities for assessing the quality of these drugs.

## Background

Malaria is a human infection caused by some protozoan parasites of the genus *Plasmodium* [1]. Many species are known to infect humans, and the most dangerous is *Plasmodium falciparum,* responsible for the most severe and fatal cases [2, 3]. Recognized as a serious public health problem worldwide, 3.3 billion people are at risk of being infected with malaria and developing disease. In 2013, 198 million cases of malaria were recorded, and the disease led to between 367,000 and 755,000 deaths [3].

Treatment is based on inhibiting the life cycle of the parasite. Currently, in order to increase the spectrum of therapeutic action and its effectiveness, as well as preventing anti-malarial drug resistance, the World Health Organization (WHO) recommends that artemisinin-based combination therapy (ACT), such as artesunate (ARS) and mefloquine (MFQ), be used for the treatment of uncomplicated *P. falciparum* malaria [3, 4]. Its mechanism of action involves the interruption of the erythrocytic schizogony cycle, destruction of latent forms of the parasite in the tissue cycle, and stopping the growth of the sexual form [5]. ACT consists of the use of two drugs with different modes of action, combining the immediate effect of an artemisinin-derivative that rapidly clears asexual blood-stage parasites and gametocytes, as well as a drug that has a longer half-life, thus eliminating residual parasites [6].

**Fig. 1:**
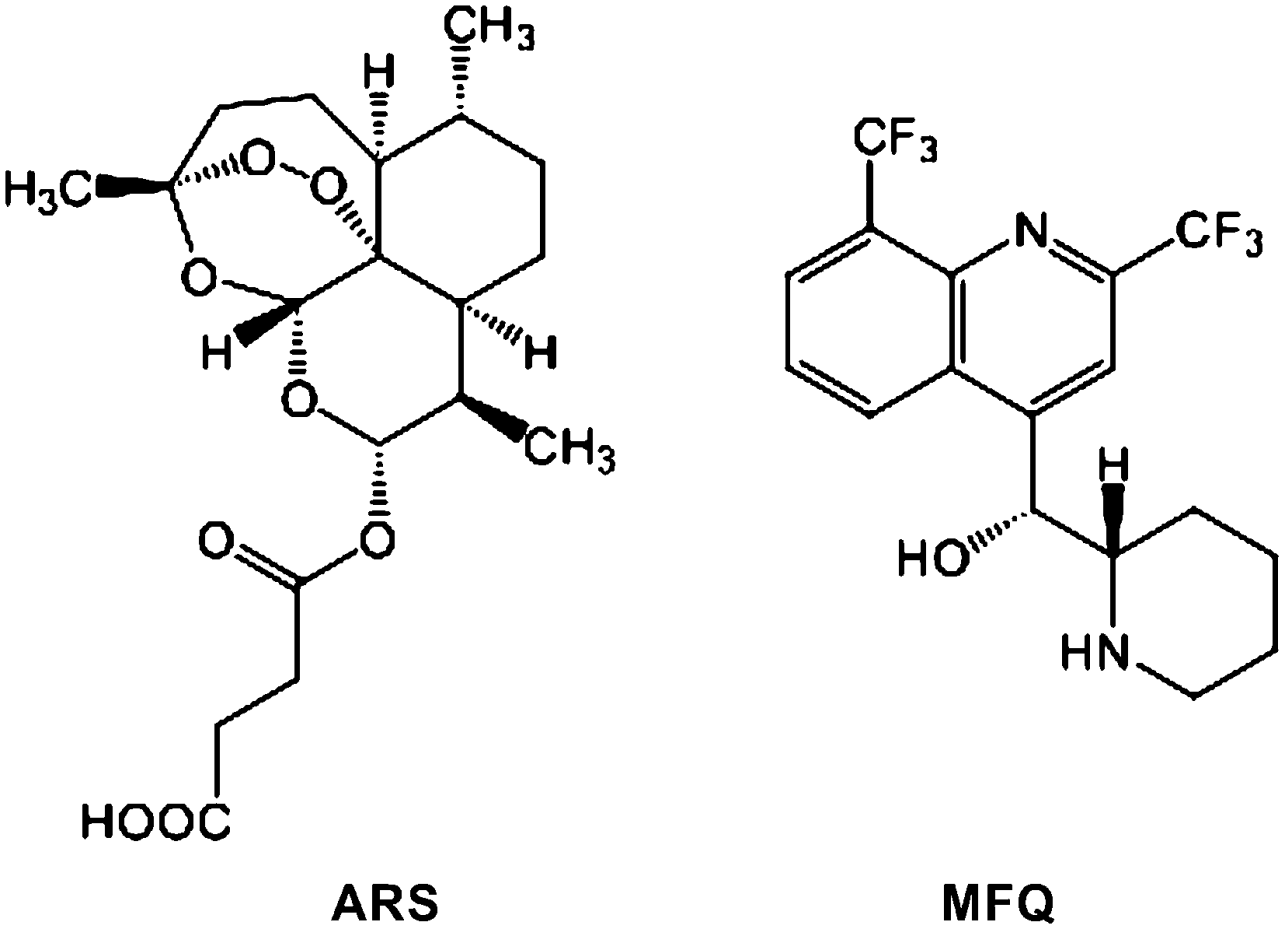
Chemical structures of artesunate (ARS) and mefloquine (MFQ)

ARS-MFQ fixed-dose combination tablets are the most common ACT treatment of uncomplicated falciparum malaria in Brazil [7]. Two kinds of tablet are currently available: one containing 25 mg of ARS and 55 mg of MFQ (corresponding to 50 mg of mefloquine base), and one containing 100 mg of ARS and 220 mg of MFQ (corresponding to 200 mg of mefloquine base) (Fig. 1).

Anti-malarials are among the most widely taken drugs in tropical countries, and have been particularly targeted by counterfeiters. The use of counterfeit drugs or sub-standard anti-malarial drugs can increase instances of morbidity, adverse effects and mortality due to excessive dose, presence of potentially toxic ingredients or pathogenic contaminants. Also, sub-therapeutic amounts of active ingredients contribute significantly to selection of resistant parasites and, thus, resistance to anti-malarial drugs [8]. Quality control of anti-malarial drug preparations may help to ensure treatment efficacy and avoid development of resistance to anti-malarial drugs [9].

Several methods for the simultaneous quantification these drugs can be found in the literature, such as ultraviolet (UV) spectrophotometry [10, 11], high performance liquid chromatography (HPLC) with UV spectrophotometry detection [12, 13], high performance thin layer chromatography (HPTLC) with UV spectrophotometry detection [13, 14], and HPLC coupled with tandem mass spectroscopy (HPLC–MS/MS) [15]. Despite being very commonly used, no methods for quality control of available products are described in the pharmacopoeias [16–19]. There are no methods using infrared (IR) associated with multivariate calibration for these drugs can be found in the scientific literature. Furthermore, this method provides direct and fast analysis, and is low cost and solvent free.

Methods based on diffuse reflectance infrared Fourier transform (DRIFT) middle infrared spectroscopy (MIR) and near infrared (NIR) spectroscopy have been adopted in the pharmaceutical industry for raw material testing and process monitoring, and have become a reliable alternative for the quality control of active pharmaceutical ingredients (APIs) [20–26]. Due to the significant overlap of IR bands, univariate techniques have limitations. However, these limitations can be circumvented through the use of multivariate calibration tools. The combination of IR spectroscopy and multivariate calibration, such as partial least squares (PLS) regression, has emerged in the last decade as a promising alternative for the quality control of APIs [27]. Furthermore, IR in combination with multivariate calibration tools has significant advantages in terms of speed, cost and reliability of analysis. Perhaps most importantly, no solvent is required by the technique.

The aim of this work was to develop and validate a simple and rapid multivariate method for direct simultaneous determination of ARS and MFQ in powdered samples of anti-malarial tablet formulations using diffuse reflectance MIR spectroscopy and PLS. The results of the multivariate method (MIR-PLS) were compared with the results obtained using HPLC–MS/MS [15].

## Methods

### Samples and chemicals

ARS, MFQ and ARS-MFQ fixed-dose combination tablets were provided by Farmanguinhos/Fundação Oswaldo Cruz (Rio de Janeiro, RJ, Brazil). The ARS standard was obtained from Farmacopeia Brasileira (>98.8 %) (Brasília, DF, Brazil), The MFQ-hydrochloride standard was obtained from Sigma-Aldrich (>98.0 %) (St Louis, MO, USA). Microcrystalline cellulose was obtained from DEG (São Paulo, Brazil), and croscarmellose sodium and magnesium stearate were obtained from Indukern do Brasil Química Ltd (São Paulo, Brazil). HPLC grade acetonitrile and methanol were purchased from Tedia (Fairfield, USA). Formic acid was purchased from Carlo Erba Reagenti, Rodano, Italy (>99.0 %). Ultrapure water was obtained using a Milli-Q purification system from Millipore (Bedford, MA, USA). The tablets had the formulation composition: 100 mg of ARS and 220 mg of MFQ (corresponding to 200 mg of mefloquine base) per tablet. The quantitative compositions of the excipients are not publicly available.

### Apparatus and software

The spectral dataset was acquired from a Bruker Alpha Fourier transform infrared (FTIR) spectrometer equipped with a diffuse reflectance accessory, and under controlled temperature (20.0 ± 0.2 °C) and humidity (45–55 %). The spectra were obtained in absorbance mode using OPUS (Optical User Software) for windows (version 6.0, Brucker Optik, Bremen, Germany). Data were handled using OPUS, MATLAB software (version 7.13, The Math-Works, Natick, USA), PLS Toolbox (version 6.5, Eigenvector Technologies, Manson, USA), and Statistica 10.0 software (StatSoft, Oklahoma, USA).

HPLC–MS/MS analyses were performed on an Agilent 1200 HPLC system (Agilent Technologies, Santa Clara, CA, USA) that consisted of a G1312B binary pump, a G1379B degasser and a G1316B column oven. This apparatus was connected with a CTC sample manager (Model 2777, Waters, Milford, MA, USA). The mass spectrometer coupled to the HPLC system was a triple quadrupole API 3200 from Applied Biosystems MDS Sciex Instruments (Foster City, CA, USA) equipped with a syringe pump (Harvard Apparatus, Holliston, MA, USA) and an electrospray (ESI) ion source. The data acquisition was performed with MS Workstation by Analyst 1.4 software (MDS Sciex, Concord, Ontario, Canada) [15].

### Experimental design

**Fig. 2:**
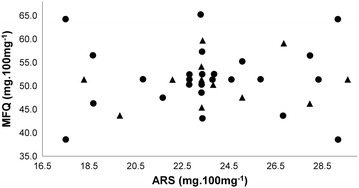
Experimental central composite design (CCD) used for variation of active pharmaceutical ingredient contents, artesunate (ARS) and mefloquine (MFQ). Samples of calibration set are represented by *circles* and validation set by *triangles*

Thirty-seven powder samples were prepared according to a central composite design (CCD) (α_rotability_ = 1.6818; α_ortogonality_ = 1.2872) with three factors (ARS, MFQ and the majority excipient), and five levels plus a central point. The number of samples for calibration must be equal to 6 × [the number of latent variables (LV) + 1] and the number of validation samples equal to 4 × LV, according to the American Society for Testing and Materials (ASTM) recommendations, as depicted in Fig. 2 [28].

The total mass of each sample was fixed at 100.0 mg, the central point of the CCD corresponds to 23.3 mg of ARS and 51.4 mg of MFQ per 100 mg of sample. The ranges of ARS and MFQ were varied from ca. 75.0–125.0 % of their nominal contents in the ARS-MFQ fixed-dose combination tablets. In the case of the minority excipients (croscarmellose sodium and magnesium stearate), was chosen not to vary their concentrations owing their low concentrations in the formulation. For the majority excipient microcrystalline cellulose, the concentration range was sufficient to ensure a final mass equivalent to 100.0 mg of sample, as defined.

### Sample preparation

The powder samples of experimental design were weighed with an analytical balance (±0.0001 g) and manually homogenized with a pestle and mortar. The spectra of the samples were recorded from 4000 to 400 cm^−1^ as the average of 32 scans and with a resolution of 4 cm^−1^. To evaluate repeatability, six replicates of samples from the central point were also obtained. To estimate intermediate precision, the results were compared with similar replicates obtained on another day by a different analyst. Fifty spectra of the empty cell were recorded to measure the instrumental noise.

### The multivariate calibration model development

The multivariate calibration model was developed using PLS regression. Several models were built using algorithm PLS1 (models for only one API) and PLS2 (models for 2 API simultaneously), cross-validation (leave-one-out), and the combination of different pre-processing methods. Models were constructed using the entire spectrum, restricted to using the leading bands of each analyte and also through the technique of intervals PLS (iPLS). The best models were chosen based on the value of the root mean square of calibration (RMSEC) and the root mean square of prediction (RMSEP).

### Analysis of ARS-MFQ fixed-dose combination tablets

The content of ten tablets with 100 mg of ARS and 220 mg of MFQ (corresponding to 200 mg of mefloquine base), was crushed and mixed into a homogeneous powder. The MIR spectra of these samples were acquired in the same conditions described in section sample preparation, in triplicate. These same samples were also analysed by HPLC–MS/MS.

### HPLC–ESI–MS/MS analysis

The analytical separations were achieved on an XBridge C18 column (50 × 2.1 mm, 5 μm) coupled with an XBridge C18 guard column (10 × 2.1 mm, 5 μm) maintained at 25 °C. The mobile phase consisted of water/acetonitrile/methanol (30:35:35 v/v/v) containing 0.1 % formic acid. The flow rate was 350 μL min^−1^ and the injection volume was 5 μL. The ESI source was operated in positive mode under the following working conditions: ion spray voltage of 5500 V; source temperature of 400 °C; nebulizer and dryer gas (nitrogen) of 40 psi; collision activated dissociation gas (CAD) of 10 psi and curtain gas (CUR) of 10 psi. Quantification was performed in multiple reaction monitoring (MRM) mode, maintaining a dwell time of 350 ms. A mass equivalent to one quarter of the tablet was subjected to extraction and mixed with internal standard, diluted in mobile phase in a volumetric flask according to the appropriate concentration. All samples were prepared under low light exposure and filtered through a 0.22 µm PVDF syringe filter prior to injection.

## Results

### Selection of excipients and experimental design

As the quantitative levels of the excipients in the ARS-MFQ fixed-dose combination tablets was not known, due to lack of access to information from the manufacturer, it was suggested a formulation based on the qualitative levels of excipients described in the leaflet and pharmacotechnical study to build a multivariate calibration model. The formulation set consisted of microcrystalline cellulose (19.5 %-diluent), sodium croscarmellose (3.5 %-disintegrating agent) and magnesium stearate (0.5 %-lubricant).

### Multivariate calibration model

**Fig. 3:**
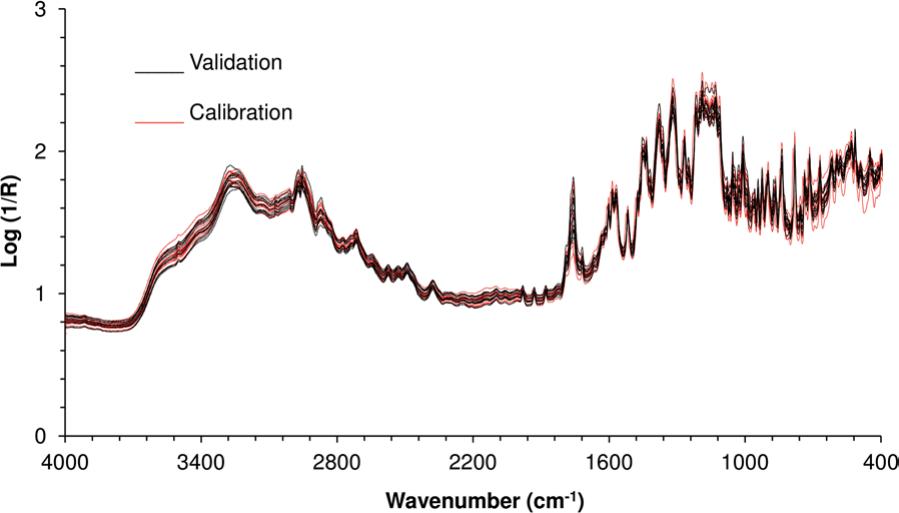
Diffuse reflectance MIR spectra of all the 37 samples of experimental design. Samples of calibration set are represented by *red spectra* and validation set by *black spectra*

The spectra of all 37 prepared samples (Fig. 3) were divided into 25 for the calibration set and 12 for the validation set, according to an experimental design (Fig. 2). The validation samples were selected in order to test the predictive ability of the model by ensuring a homogeneous and representative distribution of the two analytes within the analytical ranges. The use of preprocessing techniques in PLS model development by reflectance IR of powder samples is almost mandatory, due to the presence of non-linear baseline deviations. In this work, the most commonly used preprocessing techniques, such as multiplicative scatter correction (MSC), standard normal variate (SNV), and Savitzky-Golay first derivative with smoothing, were tested [29].

The best model was MIR-PLS using full spectra (4000–400 cm^−1^) with first derivative followed by Savitsky-Golay smoothing, followed by MSC (15 points in filter and first order polynomial fit), and mean centring (X block). The Y block utilized only mean centring. The PLS model was selected by random sub-sets cross-validation with three LVs (recommended as the minimum number of calibration and validation samples for infrared multivariate quantitative analysis). This number of LVs was used to estimate the smallest prediction error variance without significant loss of data (root mean square errors of cross validation (RMSECV × LV) [28].

Outliers may adversely affect the predictive ability of the model and therefore were investigated by analysis of the studentized residuals versus leverage, with limits of ±2.5 and 3 LV/n, respectively, considering a confidence limit of 95 % [28]. The values of studentized residual were between ±2.5 %, and values of leverage were less than 0.36. Outliers were not found. This model accounted for 89.91 % of the total variance in the X block and 80.98 % in the Y block. The averages the RMSEC, RMSECV and RMSEP values for ARS and MFQ are given in Table 1.

**Table 1:** Parameters estimated for validating the developed MIR-PLS method

Figures of merit	Parameter	Values	
		ARS	MFQ
Precision	RSD repeatability^a^	2.73	2.89
	RSD intermediate^a^	3.12	2.79
Trueness	RMSEC^b^	1.58	2.84
	RMSECV^b^	2.10	3.62
	RMSEP^b^	0.87	1.93
Range^b^		17.49–29.59	38.56–65.23
Selectivity^a^		28.70	34.40
SA (γ)^b^		25.47	35.25
γ^−1b^		0.04	0.03
RPD^c^	Calibration	1.56	1.89
	Validation	3.67	2.58
*Bia*s		−0.03	0.56
LOD^b^		0.13	0.09
LOQ^b^		0.39	0.28

^a^Per cent

^b^mg/100 mg

^c^Dimensionless units

### Validation of the multivariate model

Once the model was developed, its analytical validation is essential. For the validation process, it was considered the figures of merit recommended for multivariate calibration models [28], and national and international recommendations [30–32]. All the multivariate figures of merit estimated for this validation are listed in Table 1.

The precision, defined as the closeness of measurements of the same concentration, was assessed at two levels, based on the relative standard deviation (RSD) for six replicates of sample at 100 % of nominal concentration. The RSDs for repeatability (intra-run precision) were 2.73 and 2.89 % for ARS and MFQ, respectively, while for intermediate precision (inter-run) they were 3.12 and 2.79 %, ARS and MFQ, respectively. All RSD values are less than 5 %, in accordance with Brazilian regulations [30]. The average trueness was evaluated through the parameters RMSECV, RMSEC and mainly, RMSEP. The estimated RMSEP for the analytes were 0.87 and 1.93 mg/100 mg for ARS and MFQ, respectively. These results for trueness and precision confirm that the method is accurate.

**Fig. 4:**
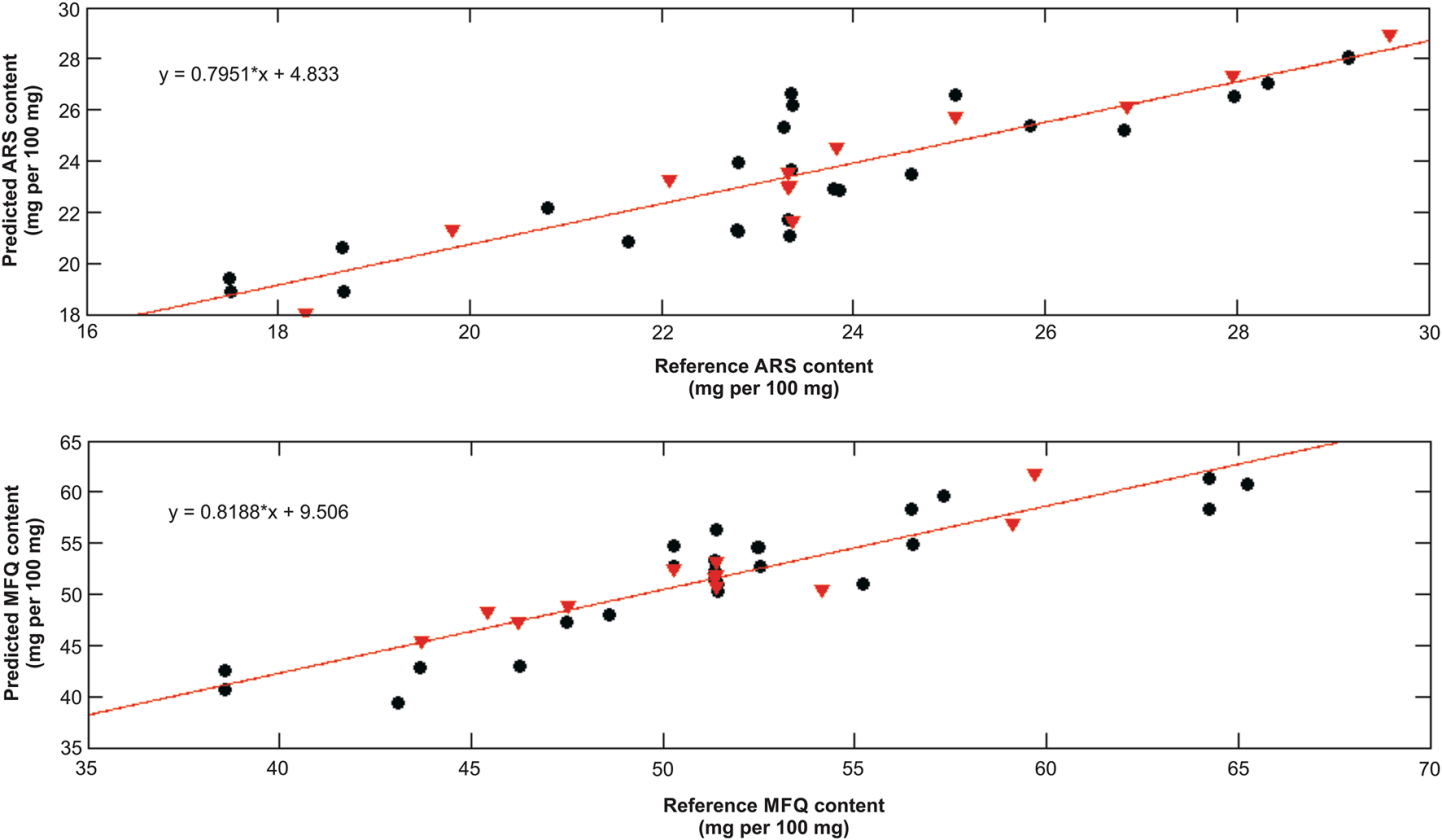
Plots of reference content versus predicted content of artesunate (ARS) and mefloquine (MFQ) determined by multivariate calibration model. Samples of calibration set are represented by *black circles* and validation set by *red down triangles*

**Fig. 5:**
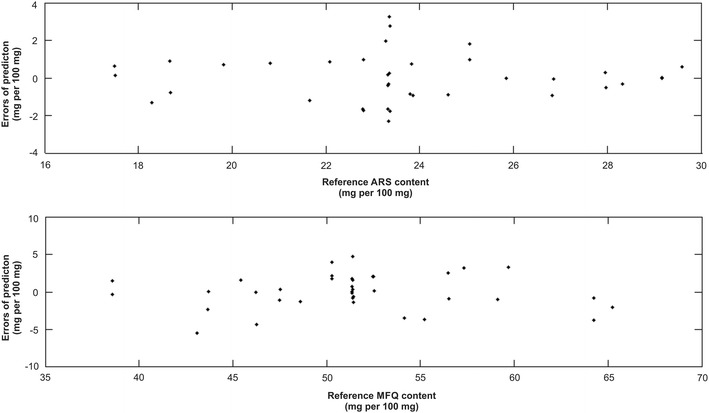
Plots of reference content versus PLS residuals for the predictions of artesunate (ARS) and mefloquine (MFQ)

The linearity was evaluated through the plot of the reference *versus* predicted values, as shown in (Fig. 4), and by verifying the random behaviours of the residual distributions for both analytes, as can be seen in Fig. 5. The correlation coefficients (r) were 0.90 for ARS and 0.91 for MFQ.

Considering the linearity and accuracy evaluations, the working ranges of this method are 17.49–29.59 mg/100 mg for ARS, and 38.56–65.23 mg/100 mg for MFQ.

The figures of merit selectivity (SEL), sensitivity (SEN), analytical sensitivity (γ), and limits of detection (LOD) were estimated based on the concept of net analytical signal (NAS), as described in the appropriate literature [22, 33–35]. The interpretation of SEL for multivariate methods is different from univariate methods, and has no practical application for quality control purposes. The SEL definition is only useful within a certain group of samples of similar composition, and for the developed method it was estimated that about 28.70 and 34.40 % of the analytical signal were used for measuring ARS and MFQ, respectively.

Since the pure SEN is not appropriate for comparison with other methods, their values were divided by the estimative of the instrumental noise (ε = 3.7 × 10^−3^) and the more useful γ (analytical sensibility) was estimated to be 25.47 and 35.25 mg/100 mg for ARS and MFQ, respectively. The inverse of γ indicated that the method was able to discriminate minimum content differences between 3.9 × 10^−2^ and 2.8 × 10^−2^ mg/100 mg for ARS and MFQ, respectively, considering random instrumental noise as the only source of errors.

Although not necessary for this kind of method, LOD and limit of quantification (LOQ) were also estimated based on the ε. The bias should be estimated only for the validation samples, and the values in Table 1, along with their standard deviation, were used in *t* tests with 11 degrees of freedom and at 95 % confidence level. The estimated *t* values were all bellow the critical *t* value (2.201), demonstrating the absence of systematic errors in the model. The residual prediction deviation (RPD) [35] is the ratio of natural variation in the samples to the size of probable errors occurring during the prediction, and it is more useful for comparing models on different data sets or in absolute terms. It was calculated for the calibration and validation sets, and the minimum RPD was estimated as 1.56 and 3.67 for ARS and MFQ, respectively (for prediction in the calibration set), and 1.89 and 2.58 for ARS and MFQ, respectively (for prediction in the validation set).

### Analysis of ARS-MFQ fixed-dose combination tablets

The developed method was applied to the simultaneous quantification of ARS and MFQ in tablets (Farmanguinhos, RJ, Brazil) and the results were compared with those obtained using HPLC–MS/MS. The same samples were analysed by the two methods in triplicate. The predicted mean values and their standard deviations are shown in Table 2. According to the *t* test, there were no significant differences between the predictions of the developed MIR-PLS method and HPLC–MS/MS, for both analytes (p > 0.05), at 95 % confidence level (all estimated *t* values were below the critical *t* = 2.776). Therefore, it can be inferred that the results are statistically equivalent between the techniques.

**Table 2:** Comparison of contents of ARS and MFQ in tablets by MIR-PLS and HPLC–MS/MS (*n* = 5)

	Mean values ± dp (n = 3)^a^		*P value* ^*b*^
	MIR-PLS	HPLC–MS/MS	
ARS	23.40 ± 0.50	22.84 ± 0.55	0.258
	22.78 ± 0.43	22.24 ± 0.46	0.220
	23.73 ± 0.27	22.77 ± 0.55	0.054
	23.20 ± 1.52	21.55 ± 0.16	0.135
	22.52 ± 0.11	22.63 ± 0.29	0.550
MFQ	49.15 ± 1.72	50.11 ± 0.75	0.428
	53.77 ± 1.91	51.17 ± 0.89	0.100
	51.67 ± 1.31	51.06 ± 0.22	0.471
	54.14 ± 3.59	52.90 ± 0.28	0.584
	52.16 ± 2.55	50.26 ± 0.13	0.266

^a^mg/100 mg of tablet

^b^t test non-paired, at 95 % confidence level

It is worth mentioning that the MIR-PLS method was developed based on a formulation proposed by this study. Even considering the possible difference between the formulation proposed and samples obtained from Brazilian healthcare units, the method showed predictive capacity equivalent to the HPLC–MS/MS method.

## Conclusions

This study was the first report of simultaneous quantification of ARS and MFQ in pharmaceutical formulations tablets by diffuse reflectance MIR spectroscopy using multivariate calibration. The developed method was validated and shown to be a suitable technique to quantify these anti-malarial agents in pharmaceutical preparations, provided results statistically similar to those obtained by HPLC–MS/MS, and may be successfully employed for quality control analysis. ARS and MFQ tablets analysed by the validated method showed adequate quality and drug contents in agreement with the labelled amounts. Compared to liquid chromatography methods, the MIR-PLS method has advantages such as its low cost, rapidity and ability to provide direct solvent-free analysis. It may be considered suitable for routine quality control determinations.

## Abbreviations

ACT: artemisinin-based combination therapy
API: active pharmaceutical ingredient
ARS: artesunate
ASTM: American Society for Testing and Materials
CAD: collision activated dissociation gas
CCD: central composite design
CUR: curtain gas
DRIFTS: diffuse reflectance infrared Fourier transform spectroscopy
ESI: electrospray
FTIR: Fourier transform infrared
HPLC–MS/MS: high performance liquid chromatography coupled with tandem mass spectroscopy
HPTLC: high performance thin layer chromatography
iPLS: interval partial least squares regression
IR: infrared
LOD: limits of detection
LOQ: limit of quantification
LV: latent variable
MFQ: mefloquine
MIR: middle infrared spectroscopy
MIR-PLS: middle infrared spectroscopy and partial least squares regression
MRM: multiple reaction monitoring
MSC: multiplicative scatter correction
NAS: net analytical signal
NIR: near infrared spectroscopy
PLS: partial least squares regression
RMSEC: root mean square of calibration
RMSEP: root mean square of prediction
RMSECV: root mean square of cross-validation
RPD: residual prediction deviation
RSD: relative standard deviation
SEL: selectivity
SEN: sensitivity
SNV: standard normal variate
UV: ultraviolet
WHO: World Health Organization
γ: analytical sensitivity
